# Dental Treatment Characteristics of Autistic Children and Differences in Dental Procedures under General Anesthesia Relative to Healthy Counterparts

**DOI:** 10.3390/children10030466

**Published:** 2023-02-26

**Authors:** Bayan Alghafis, Abdulaziz Alharbi, Sanaa Al-Haj Ali, Faisal Alsineedi, Ohoud Alsudairi

**Affiliations:** 1 College of Dentistry, Qassim University, Qassim 52571, Saudi Arabia; 2 Dental Department, King Fahd Military Medical Complex, Dhahran 31932, Saudi Arabia; 3 Department of Orthodontics and Pediatric Dentistry, College of Dentistry, Qassim University, Qassim 52571, Saudi Arabia

**Keywords:** autism spectrum disorder, behavior, dental care for children, general anesthesia

## Abstract

Here, we assess the dental treatment characteristics among children with autism spectrum disorder (ASD) and compare the dental procedures delivered under general anesthesia (GA) with those of healthy-matched counterparts. In this retrospective cohort study, we collected data from medical records of ASD children (*n* = 82) which included demographic profile, medical status (including the severity of the ASD and associated comorbidities), and dental history (including dental visits, behavior, care approach, and dental procedures provided). For those children who received dental GA (DGA) (*n* = 64), we compared all procedures delivered and the number of repeat sessions with healthy children (*n* = 64). Our results reveal that most ASD children (78%) received DGA, mainly with one repeat session (63.4%). The dental procedures provided to the children differed significantly according to the severity of the ASD and the behavior of the children. Furthermore, increasing severity led to significantly worse behavior of the children, increased need for DGA and repeat sessions. Comparison of the dental procedures under GA with healthy children revealed a significantly lower mean of almost all dental procedures in ASD children, except fissure-sealed teeth (*p* < 0.05). Considering these findings, DGA is unavoidable for ASD children with moderate-to-severe conditions or negative behavior in the dental office, even when their dental needs are lower than healthy children. The severity of ASD was the most important factor affecting the behavior of the children and the care approach they received. Along with children’s behavior, they most influenced the dental procedures delivered and the need for repeat DGA.

## 1. Introduction

Autism spectrum disorder (ASD) is a complex developmental condition that involves persistent challenges in social interaction, speech, nonverbal communication, and repetitive behavior [1]. The signs of this condition show early, during the first three years of life [2], and some comorbidities may coexist with it such as sensory abnormalities, motor deficits, cognitive deficits, epilepsy, and psychiatric diagnoses [3].

Several studies have investigated the oral health status of children with ASD with conflicting results regarding dental caries experience [4,5,6,7,8]. Most studies agree that those children have worse oral hygiene and greater periodontal needs than healthy children [4,6,7,8]. However, their caries experience ranged from being worse or similar to healthy children [4,7,8] to being better than healthy children [6].

ASD children are at risk for dental diseases as well as prone to unmet dental needs [9], particularly those with an intellectual disability or greater behavioral difficulties [10]. Those children create difficulties for their parents by resisting oral hygiene measures at home [11], and often behave negatively in the dental office, restricting behavioral management strategies [9].

The barriers to dental care that children with ASD face were assessed primarily from parental reports [9,10,11,12,13,14,15]. Cost of the treatment [9], family income or socioeconomic status [13], finding a dentist with the skills or willingness to treat the child [9,11,12,13,15], the behavior of the child [9,12,13], the presence of an intellectual disability, the lack of an established medical home [10], and the inability to travel to the dental surgery [10,11] were all identified as factors mitigating against receiving optimum care. However, proxy reporting by parents or caregivers is subject to recall bias and to the willingness to share the information [16]. Furthermore, the potential for inaccuracy of ASD diagnosis in parental reports exists [9,10,14].

A small number of studies have retrospectively analyzed the dental needs and the care approach delivered to children with ASD, with a particular emphasis on the need for dental general anesthesia (DGA) [17,18,19,20]. According to Mangione et al. [17], nitrous oxide/oxygen inhalation with or without premedication was efficient in children with ASD <12 years of age and allowed conservative procedures to be undertaken in 77% of the children. However, most studies agree that DGA was the dominant care approach for ASD children or adolescents [17,18,19,20]. Parry et al. [18] and Azimi et al. [19] even emphasized the frequent need for repeat DGA for those children.

Objective assessment of the dental treatment characteristics of ASD children including their dental visits, behavior during those visits, the adopted care approach or dental procedures delivered to them, and associated demographic factors were insufficiently addressed outside the scope of parental reports. Furthermore, they were not assessed previously in Saudi Arabia. Recently, Azimi et al. [20] noted differences in the dental procedures delivered to children with ASD under general anesthesia (GA) from those without the condition without specifying whether the comparison group included healthy children, those with other special health care needs, or both. On the other hand, Arnold et al. [21] stated briefly that children with ASD received significantly fewer pulpotomies and crowns than healthy children under GA. An in-depth comparison of the dental procedures that children with ASD receive under GA compared with healthy children is essential since it may detect a variation in dental needs, treatment, or strategic planning for ASD children. Therefore, this study aimed to assess the dental treatment characteristics of children with ASD and compare the dental procedures delivered to them under GA from those of healthy-matched counterparts

## 2. Materials and Methods

### 2.1. Subjects and Ethical Approval

A total of 82 children diagnosed with ASD (up to 18 years of age) with varying degrees of severity and 64 healthy uncooperative children who matched the ASD children for gender, dentition stage, family economic level, and health insurance coverage all presented for dental treatment at king Fahd military medical complex, Dhahran, Saudi Arabia during the period January 2018–January 2022 were included in this retrospective cohort study. All children had an ASA physical status classification of either one or two.

To determine the severity of ASD, the Diagnostic and Statistical Manual of Mental Disorders (DSM-5) classification by the American Psychiatric Association was followed, where a mild disease stood for the first level: “Requiring support”. A moderate disease stood for level 2: “Requiring substantial support”, and a severe disease stood for Level 3 “Requiring very substantial support” [1]. This study was carried out by medical record review. The caregivers of the children were contacted and informed consent was obtained verbally. Furthermore, ethics approval was obtained from the Armed Forces Hospitals Eastern Region Institutional Review Board (IRB) (protocol # AFHER–IRB-2022-23, issued on 30 May 2022) before the study was conducted and the methods employed were approved. Moreover, all procedures adhered to the tenets of the Declaration of Helsinki and all relevant local regulations.

### 2.2. Data Collection

#### 2.2.1. ASD Children

For children with ASD the following data were collected: (1) the demographic profile including the patient’s gender, family economic level (low, middle, high), health insurance coverage (yes/no) and dentition stage (primary/mixed or permanent); (2) the medical status of the child including severity of the ASD (mild/moderate/or severe) and associated comorbidities (one or more condition (yes/no)); and (3) the dental history of the child including previous dental visits (yes/no), behavior in previous visits according to Frankl behavior rating scale [3] ((rating 1: definitely negative behavior (−−), rating 2: negative behavior (−), rating 3: positive behavior (+), and rating 4: definitely positive behavior (++)), the care approach in previous visits (no treatment, simple treatment without local anesthesia, simple treatment under local anesthesia, treatment using nitrous oxide sedation, treatment using protective stabilization (active/passive), and treatment under GA), the patient’s behavior at presentation, the care approach provided to the patient at the hospital and the dental procedures delivered—including the number of restorations, extractions, teeth receiving any form of pulp therapy, crowned teeth, fissure sealed teeth, and other treatments (e.g., dental prophylaxis, space maintainers, scaling, surgery among others). Furthermore, the number, reason (dental caries with pain, molar incisor hypomineralization, dental trauma, supernumerary teeth, oral pathology, orthodontic extraction, surgical extraction, amelogenesis imperfecta, dentinogenesis imperfecta, surgical extraction, other), and time interval of all repeat DGA sessions during the study period were collected.

#### 2.2.2. Healthy Children

A total of 64 healthy children who matched the ASD children for demographic profile were included (39 boys and 25 girls). Those children were uncooperative in the dental chair and consequently received DGA in the hospital during the study period (Frankl rating scale one (−−) or two (−)). For those children, the number of all dental procedures performed under GA was recorded. Furthermore, the number, reason, and time interval of repeat DGA sessions, if required, during the selected time interval was collected. All DGA sessions in the hospital were conducted with naso-endotracheal intubation by senior pediatric dentists and in case of an uneventful procedure, the children were discharged two hours after recovery.

### 2.3. Data Analysis

All calculations and tests were performed on Excel (Microsoft Office^®^) and the Statistical Package for the Social Sciences (SPSS) computer software (Version 22, Chicago, IL, USA). Descriptive statistics were performed to determine the demographic profile of ASD children, their medical status, and their dental history. A chi-squared test was used to determine the association of the ASD children’s demographic profile and medical status with dental history findings. Furthermore, differences in the dental procedures according to whether or not the children had previous dental visits and their behavior during the visits were determined. In case the children received DGA, the association between the number of sessions delivered to ASD children and their demographic profile, medical status, and behavior was determined. Non-parametric Mann–Whitney U and Kruskal–Wallis tests were used to determine the differences in the dental procedures delivered to the children according to their demographic profile, medical status, and dental history findings, as well as differences from healthy children. Dunn’s (1964) procedure with a Bonferroni correction was used for pair-wise comparisons when the Kruskal–Wallis tests were significant. Probability values of *p* < 0.05 were considered statistically significant.

## 3. Results

The demographic characteristics of ASD children, their medical status details, and dental history findings are summarized in Table 1. Children who had comorbidity represented 41.5% with attention deficit hyperactivity disorder (ADHD) and epilepsy/seizures being the most common comorbidities (25.6%). Other less common comorbidities included hypothyroidism/asthma (7.3%), down syndrome/cleft lip, and cleft lip and palate (7.3%). Almost all ASD children (92.7%) had previous dental visits, with the majority (74.4%) showing negative behavior in previous visits as well as at presentation in the hospital (75.6%). The adopted care approach was mostly DGA (67.1% in previous visits vs. 78% in the hospital). Teeth extractions and other treatments, mostly comprising dental prophylaxis, followed by restorations, were the most frequent procedures (86.6%, 78%, and 65.9%, respectively). The age range for children with ASD was between 3 and 13 years while it was between 3 and 10 years for healthy children. All children had health insurance coverage.

Table 2 and Table 3 show the association of the children’s demographic profile and medical status with the dental history findings. A statistically significant association was found between ASD severity and behavior and care approach in previous visits and at presentation (*p* < 0.05), where all ASD children with severe conditions behaved negatively or definitely negatively in previous visits and at presentation compared with those with mild conditions (50%). Furthermore, the great majority of children with moderate-to-severe conditions received treatment under GA compared with those with mild conditions. Furthermore, a statistically significant association was found between family economic level and behavior in previous visits (*p* = 0.038). Just 53.8% of children from high-economic families behaved negatively in previous visits compared with 76.5–80% of children from low- and middle-economic families.

Differences in the dental procedures delivered to ASD children according to their demographic profile, medical status, and dental history findings are shown in Table 4, Table 5 and Table 6. The findings from Table 4 show that ASD children in the primary dentition had a significantly higher mean of crowns (*p* = 0.001), and boys had a significantly higher mean of fissure-sealed teeth (*p* = 0.032). Additionally, the mean of extractions was significantly different according to the family’s economic level (*p* = 0.048), while the mean of restorations and other treatments was significantly different according to the ASD severity (*p* = 0.002 and *p* = 0.01, respectively) (Table 5). Table 6 shows that the mean of restorations, fissure-sealed teeth, crowns, and other treatments was significantly different according to the behavior in previous visits and at presentation (*p* < 0.05), where children who behaved negatively or definitely negatively in previous visits and at presentation had a significantly higher mean of restorations and other treatments than those who behaved positively. Furthermore, the children whose behavior was definitely negative at presentation had a significantly higher mean of crowns and fissure-sealed teeth, whereas children who presented as a first visit had a significantly higher mean of fissure-sealed teeth, crowns, as well as other treatments than those who behaved positively at presentation.

**Figure 1:**
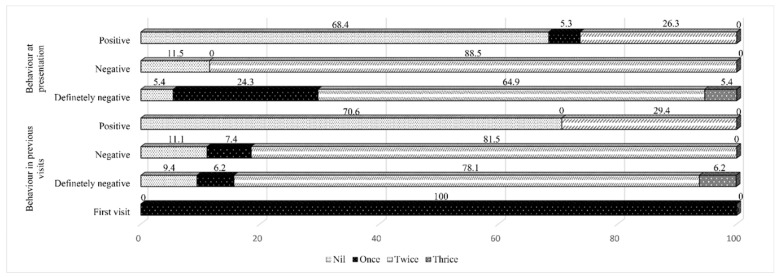
The dental general anesthesia sessions provided to children with autism spectrum disorder according to their behavior in the dental visits.

The number of DGA sessions provided to the children was in the following order: nil (22%), one session (12.2%), two sessions (63.4%), and three sessions (2.4%). Analysis of the factors which had an association with the number of DGA sessions delivered for ASD children revealed an association with the severity of the disorder (*p* = 0.029) (Table 7). Furthermore, there was an association with the behavior of the child both in previous visits and at presentation (*p* < 0.0001) (Figure 1). The reason for the repeat DGA sessions was “dental caries with pain”. Only two children with ASD had a repeat DGA in the same hospital and that was three years after the initial session.

**Figure 2:**
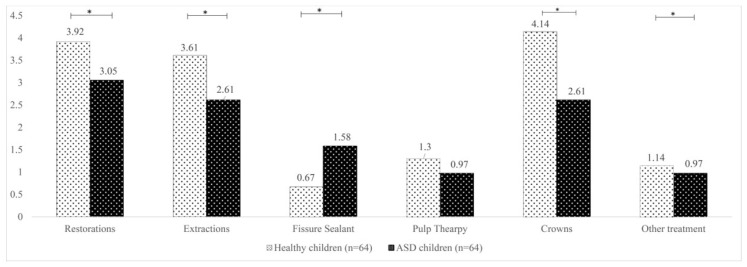
Comparison of the dental procedures under general anesthesia between children with autism spectrum disorder and healthy-matched children (mean values are displayed). * Statistically significant.

When the dental procedures delivered to ASD children under GA were compared with those of healthy-matched counterparts who also received DGA, children with ASD were found to have a significantly lower mean of teeth restorations (*p* = 0.012), extractions (*p* = 0.013), crowns (*p* = 0.001), as well as other treatments (*p* = 0.002). However, they had a significantly higher mean of fissure-sealed teeth (*p* = 0.002) (Figure 2). No repeat DGA sessions were performed for healthy children during the study period.

## 4. Discussion

In the present study, ASD severity influenced children’s behavior and care delivery. It also affected the number of DGA sessions needed. All children with severe ASD behaved negatively compared with those with mild conditions (50%). Additionally, more children with moderate-to-severe conditions received DGA and repeat sessions than those with mild conditions. Similarly, Azimi et al. [20] reported that children with severe ASD and intellectual disability were much more likely to be hospitalized than those with mild-to-moderate conditions. Children with severe conditions in this study comprised 20% of ASD children. Hence, it is no surprise that those children received DGA and repeated treatments because of their negative behavior. GA in the operating room creates a controlled environment where care is delivered efficiently and effectively for children with behavior problems [4]. The repeat DGA sessions in the present study were at least three years after previous sessions, which is consistent with safety regulations concerning avoiding repeat sessions within two years [22].

What seemed concerning in the present study is the great reliance on DGA as a caring approach for ASD children. Much more children than those with severe ASD received DGA. It has been previously shown that ASD children with mild intellectual problems and the absence of severe behavioral problems may handle dental treatment under local anesthesia and, when necessary, nitrous oxide–oxygen sedation [23]. In this study, the percentage of children who accepted dental treatment in the dental chair was almost the same whether inside or outside the hospital (22–24%). Teeth extraction was the most performed procedure in the dental chair, and one or two children received a form of pulp therapy. Pulp therapy treatments are considered time-consuming and require compliance, compared with tooth extraction, which is a quick procedure, perhaps done in an emergency, to relieve the child’s pain [17].

A finding supporting the need for the high rate of DGA in this study is the negative behavior of most of the children (70–75%). A significantly greater number of repeat DGA sessions was also noted among children with negative or definitely negative behavior. Difficult behavior in the dental office was significantly related to the inability to deliver dental care in the dental chair for ASD children [8,13]. It is also likely that parents will accept a behavioral management approach if they have experienced it before with their child [3]. In this study, more than two-thirds of the children (67%) experienced DGA in previous visits and had health insurance coverage, so they had no cost issues associated with DGA. Additionally, previous reports have pointed out increased acceptance of DGA by parents of ASD children than other advanced behavior management techniques such as protective stabilization or sedation [3,4,23], most likely due to concerns about the negative systemic effects of nitrous oxide, or fear of drug interactions, especially for children with associated comorbidities who take regular medications which can interact with or jeopardize the success of the sedation regimen [23]. Nevertheless, DGA is a costly service for children who lack health insurance; it requires more staff, specialist facilities, and equipment for safe provision [18]. Furthermore, DGA carries risks, especially when the child gets exposed to repeat treatments. Given that pediatric dentists provide dental services to children with ASD at King Fahad Hospital, for future studies it is crucial to assess the views of pediatric dentists in the region in terms of the barriers they face while treating children with ASD, including the availability of trained dental personnel/physicians to assist them with managing those children, and the practices which are followed to overcome communication challenges. Weil et al. [24] have previously reported that behavior management strategies chosen by dental care providers might not be the most beneficial strategies when managing patients with ASD. Furthermore, unfamiliarity with comprehensive protective stabilization by physicians and the inability to manage children with difficult behavior has been previously reported [25].

Another finding in this study was the association between the family’s economic level and children’s behavior in previous visits. A greater proportion of those from low- or middle-economic families (76.5–80%) behaved negatively compared with those from high-economic levels (53.8%). However, the same finding was not observed at the hospital. A noted rise in positive behavior of children from a middle economic level resulted in the loss of this significant difference. In this context, John and Ausderau [26] noted that children from higher economic levels had increased functional independence behavior compared with those from lower levels. This perhaps explains the noted association. Furthermore, dental treatments were provided to the children in the hospital by pediatric dentists in this study, who are known for their greater willingness and skills in managing ASD children than general dentists [24]. This might be the reason behind the rise in positive behavior of children from middle economic families.

Regarding dental procedures delivered to ASD children, teeth extractions and other treatments, mostly dental prophylaxis, followed by restorations, were the most frequent (86.6%, 78%, and 65.9%, respectively). Previous reports have reported the same [3,17,18,19]. However, certain influencing factors were observed. For example, children in the primary dentition had a significantly higher mean of crowns. This should be expected, as primary teeth in children in the mixed/permanent dentition are either exfoliated or would be exfoliated in a few years; hence, the need for crowns may be converted to tooth extraction [27,28]. Another finding was that children of low-economic families had a significantly higher mean of extractions. It could be that those children had more un-restorable teeth, although no data were available in the children’s records to confirm this.

Furthermore, children with mild ASD severity had a significantly lower mean of restorations and other treatments, while those who behaved positively had the least mean of most dental procedures. Dangulavanich et al. [29] have previously reported that ASD children who behaved positively were 15 times more cooperative during dental treatment than those who behaved negatively. It is reasonable to assume that those children similarly cooperate with their caregivers in performing oral hygiene measures. Consequently, their dental needs would be less than those with difficult behavior.

When the dental procedures delivered under GA were compared between ASD children and those of healthy matched counterparts, ASD children were found to have a significantly lower mean of restorations, extractions, crowns, and other treatments but significantly higher mean of fissure-sealed teeth. This could reflect a greater emphasis on preventive treatments among ASD children, particularly after considering the high percentage of negative behavior in the studied population. Arnold et al. [21] similarly found that ASD children underwent fewer dental procedures under GA than healthy children, as healthy children who are scheduled for GA due to lack of cooperation generally have extensive dental work required. On the other hand, ASD patients are scheduled for GA due to their inability to have even simple dental procedures performed in the office. Consequently, the oral health status or dental needs of ASD children are not necessarily inferior to healthy children [6,30], particularly when considering that ASD children live under an increased level of supervision than healthy children and adolescents and are perhaps under better control concerning their food choices.

This study has strengths and limitations that should be addressed. This study was the first of its kind in Saudi Arabia. It was conducted in a hospital with a leading autism center; hence the diagnosis of ASD and severity rating are more objective and reliable than those obtained through questioning the parents of the children. While some may claim that having health insurance coverage could have resulted in a greater dependence on DGA than the required need, it is important to remember that those who have health insurance are more likely to seek dental services and have a dental history than those without insurance [14]. Furthermore, despite the fact that GA is an offered advanced behavior management option for all children with behavior challenges regardless of their health insurance status, parents of uninsured children will not give consent to proceed with GA due to the inability to afford its expenses. Additionally, the study’s retrospective nature and the relatively small sample from a single hospital can hinder the generalizability of the results and constitute limitations. It is worth mentioning that most retrospective investigations that analyzed the dental needs and treatments delivered to ASD children also involved small samples (79–83 children) and samples obtained from single hospitals [17,18].

## 5. Conclusions

The severity of the ASD was the most important factor affecting the children’s behavior during the dental visits and the care approach provided to them. This, along with the behavior during the dental visits, comprised the two factors that most influenced the dental procedures the children received and the number of DGA sessions.

Compared with healthy counterparts, the dental needs in terms of almost all procedures delivered under GA were lower in ASD children, except for fissure-sealed teeth, which were higher in ASD children.

DGA is unavoidable for ASD children with severe conditions or negative behavior in the dental office.

## Figures and Tables

**Table 1:** Characteristics of ASD children (*n* = 82, *n* (%)).

**Demographic Data**		
Gender	Boy	57 (69.5)
	Girl	25 (30.5)
Dentition stage	Primary	25 (30.5)
	Mixed/permanent	57 (69.5)
Family economic level	Low	34 (41.5)
	Middle	35 (42.7)
	High	13 (15.9)
**Medical Status**		
ASD severity	Mild	32 (39)
	Moderate	33 (40.2)
	Severe	17 (20.7)
Associated comorbidities (one or more)		34 (41.5)
**Dental History**		
Previous dental visits	Yes	76 (92.7)
	No	6 (7.3)
Behavior in previous visits (Frankl rating scale)	First visit	6 (7.3)
	Rating 1 (−−)	31 (37.8)
	Rating 2 (−)	30 (36.6)
	Rating 3 (+)	15 (18.3)
	Rating 4 (++)	0 (0)
Care approach in previous visits	Nil (first visit)	7 (8.5)
	Simple treatment with LA ^‡^	20 (24.4)
	Treatment under GA	55 (67.1)
Behavior at presentation (Frankl rating scale)	Rating 1 (−−)	37 (45.1)
	Rating 2 (−)	25 (30.5)
	Rating 3 (+)	20 (24.4)
	Rating 4 (++)	0 (0)
Care approach at the hospital	Simple treatment with LA **	18 (22)
	Treatment under GA	64 (78)
Dental procedures provided at the hospital	Restorations	54 (65.9)
	Extractions	71 (86.6)
	Fissure sealant	41 (50)
	Pulp therapy	32 (39)
	Crowns	41 (50)
	Other treatment ^†^	64 (78)

ASD: Autism spectrum disorder, LA: local anesthesia, GA: general anesthesia. † Dental prophylaxis (61 cases), scaling (one case), or space maintainer (one case). ‡ Extraction of one or two teeth (18 cases), pulp therapy (two cases). ** Extraction of one or two teeth (17 cases), pulp therapy/root canal treatment: one case.

**Table 2:** Association of the demographic profile of ASD children (*n* = 82) and their medical status with their previous dental visits and behavior.

Variable	Dentition Stage (Mixed/ Permanent *n*%)	Gender (boys *n*%)	ASD + Comorbidities *n* (%)	Economic Level (*n*%)			ASD Severity (*n*%)		
				Low	Middle	High	Mild	Moderate	Severe
Previous visits (% yes)									
	54 (94.7)	54 (94.7)	32 (94.1)	33 (97.1)	31 (88.6)	12 (92.3)	28 (87.5)	31 (93.9)	17 (100)
*p*-value	0.122	0.259	0.513		0.399			0.261	
Behavior in Previous visits									
First visit	2 (3.5)	3 (5.3)	2 (5.9)	1 (2.9)	4 (11.4)	1 (7.7)	4 (12.5)	2 (6.1)	0 (0)
Definitely negative	24 (42.1)	24 (42.1)	16 (47.1)	12 (35.3)	12 (34.3)	7 (53.8)	6 (18.8)	13 (39.4)	12 (70.6)
Negative	17 (28.6)	20 (35.1)	12 (35.3)	14 (41.2)	16 (45.7)	0 (0)	10 (31.2)	15 (45.5)	5 (29.4)
Positive	14 (24.6)	10 (17.5)	4 (11.8)	7 (20.6)	3 (8.6)	5 (38.5)	12 (37.5)	3 (9.1)	0 (0)
*p*-value	0.11	0.539	0.414	0.038 *			0.001 *		
Behavior at presentation									
Definitely negative	26 (45.6)	29 (50.9)	18 (52.9)	12 (35.3)	17 (48.6)	8 (61.5)	11 (34.4)	15 (45.5)	11 (64.7)
Negative	15 (26.3)	15 (26.3)	8 (23.5)	15 (44.1)	10 (28.6)	0 (0)	5 (15.6)	14 (42.4)	6 (35.3)
Positive	16 (28.1)	13 (22.8)	8 (23.5)	7 (20.6)	8 (22.9)	5 (38.5)	16 (50.0)	4 (12.1)	0 (0)
*p*-value	0.16	0.268	0.422		0.062			<0.0001 *	

* statistically significant, chi-squared test. ASD: Autism spectrum disorder, GA: general anesthesia, LA: local anesthesia.

**Table 3:** Association of the demographic profile of ASD children (*n* = 82) and their medical status with the care approach provided.

Variable	Dentition Stage (Mixed/Permanent *n*%)	Gender (Boys *n*%)	ASD+ Comorbidities *n*%	Economic Level *n*%			ASD Severity *n*%		
				Low	Middle	High	Mild	Moderate	Severe
Care approach in previous visits									
First visit	2 (3.5)	4 (7.0)	2 (5.9)	1 (2.9)	4 (11.4)	2 (15.4)	4 (12.5)	2 (6.1)	1 (5.9)
Simple treatment with LA	17 (29.8)	14 (24.6)	7 (20.6)	8 (23.5)	9 (25.7)	3 (23.1)	13 (40.6)	5 (15.2)	2 (11.8)
Treatment under GA	38 (66.7)	39 (68.4)	25 (73.5)	25 (73.5)	22 (62.9)	8 (61.5)	15 (46.9)	26 (78.8)	14 (82.4)
*p*-value	0.071	0.756	0.553		0.607			0.042 *	
Care approach at the hospital									
Simple treatment with LA	14 (24.6)	11 (19.3)	5 (14.7)	8 (23.5)	7 (20.0)	3 (23.1)	13 (40.6)	3 (9.1)	2 (11.8)
Treatment under GA	43 (75.4)	46 (80.7)	29 (85.3)	26 (76.5)	28 (80.0)	10 (76.9)	19 (59.4)	30 (90.9)	15 (88.2)
*p*-value	0.289	0.274	0.144		0.934		0.005 *		

* statistically significant, chi-squared test. ASD: Autism spectrum disorder, GA: general anesthesia, LA: local anesthesia.

**Table 4:** The dental procedures provided to ASD children according to their demographic profile.

Procedures	Dentition Stage ^†^		Gender ^†^		Economic Level ^‡^		
	Primary	Mixed/Permanent	Boy	Girl	Low	Middle	High
Restorations							
Mean (SD)	3.22 (3.20)	3.13 (2.78)	2.82 (2.96)	2.04 (2.83)	2.71 (3.14)	2.73 (2.96)	2.56 (1.38)
*p*-value	0.346		0.168		0.977		
Extractions							
Mean (SD)	3.34 (2.02)	3.01 (2.46)	2.98 (2.28)	2.33 (2.45)	3.17 ^a^ (2.93)	1.46 ^b^ (1.30)	2.74 ^c^ (1.11)
*p*-value	0.979		0.971		0.048 *		
					a vs. b: 0.043 *		
Fissure sealant							
Mean (SD)	1.66 (1.32)	1.46 (2.30)	2.39 (2.32)	0.71 (1.27)	0.92 (1.38)	2.56 (2.54)	2.14 (2.26)
*p*-value	0.535		0.032 *		0.519		
Pulp therapy							
Mean (SD)	1.31 (1.06)	1.01 (1.15)	0.65 (0.94)	1.40 (1.41)	0.79 (1.34)	0.62 (0.89)	1.47 (0.97)
*p*-value	0.089		0.468		0.863		
Crowns							
Mean (SD)	3.91 (2.49)	1.42 (2.30)	2.65 (2.42)	1.62 (2.43)	1.86 (2.62)	3.07 (2.37)	0.98 (0.75)
*p*-value	0.001 *		0.440		0.613		
Other							
Mean (SD)	2.17 (2.30)	0.67 (0.52)	0.87 (0.45)	0.71 (0.56)	0.64 (0.48)	0.73 (0.45)	1.0 (0.57)
*p*-value	0.211		0.592		0.139		

* statistically significant. † Mann–Whitney U test. ‡ Kruskal–Wallis test. Different letters indicate statistically significant pairwise comparisons. ASD: Autism spectrum disorder.

**Table 5:** The dental procedures provided to ASD children according to their medical status.

Procedures	Comorbidities ^†^		ASD Severity ^‡^		
	Yes	No	Mild ^a^	Moderate ^b^	Severe ^c^
Restorations					
Mean (SD)	2.73 (2.63)	2.44 (3.10)	1.41 (2.37)	3.48 (2.69)	2.91 (3.72)
*p*-value	0.588		0.002 *		
			a vs. b: 0.002 *		
Extractions					
Mean (SD)	1.97 (1.66)	2.78 (3.10)	1.75 (1.29)	2.76 (2.68)	4.25 (3.02)
*p*-value	0.445		0.779		
Fissure sealant					
Mean (SD)	1.52 (2.48)	1.21 (1.78)	1.00 (1.84)	1.96 (2.47)	0.66 (1.07)
*p*-value	0.576		0.556		
Pulp therapy					
Mean (SD)	0.78 (0.51)	0.68 (1.01)	0.42 (0.88)	0.72 (0.89)	2.28 (1.74)
*p*-value	0.880		0.605		
Crowns					
Mean (SD)	1.43 (2.21)	2.00 (2.52)	0.96 (2.03)	2.28 (2.07)	2.42 (3.34)
*p*-value	0.643		0.156		
Other					
Mean (SD)	0.78 (0.51)	0.68 (0.47)	0.75 (0.59)	1.03 (0.27)	0.75 (0.45)
*p*-value	0.316		0.01 *		
			a vs. b: 0.008 *		

* statistically significant. † Mann–Whitney U test. ‡ Kruskal–Wallis test. Different letters indicate statistically significant pairwise comparisons. ASD: Autism spectrum disorder.

**Table 6:** The dental procedures provided to ASD children according to previous dental visits and the children’s behavior.

Procedures	Previous Dental Visits ^†^		Behavior in the Previous Visits ^‡^				Behavior at Presentation ^‡^		
	Yes	No	First Visit ^a^	Definitely Negative ^b^	Negative ^c^	Positive ^d^	Definitely Negative ^a^	Negative ^b^	Positive ^c^
Restorations									
Mean (SD)	2.54 (2.67)	2.21 (1.46)	1.00 (1.54)	3.43 (3.01)	2.70 (2.33)	0.47 (0.94)	4.05 (2.99)	2.80 (2.33)	1.09 (1.07)
*p*-value	0.079		<0.0001 *				<0.0001 *		
			b vs. d: <0.0001 * c vs. d: 0.003 *				a vs. c: 0.001 * b vs. c: 0.001 *		
Extractions									
Mean (SD)	2.91 (2.39)	2.28 (1.49)	2.50 (1.51)	2.78 (2.84)	2.29 (2.39)	1.58 (0.79)	2.75 (2.69)	2.30 (2.44)	1.63 (0.83)
*p*-value	0.586		0.663				0.566		
Fissure sealant									
Mean (SD)	2.15 (2.23)	3.00 (2.23)	3.50 (1.97)	1.93 (2.19)	1.77 (2.60)	0.76 (1.39)	2.32 (2.26)	1.73 (2.55)	1.32 (1.33)
*p*-value	0.091		0.035 *				0.016 *		
			a vs. d: 0.031 *				a vs. c: 0.013 *		
Pulp therapy									
Mean (SD)	0.71 (1.17)	2.48 (1.13)	1.67 (1.03)	0.72 (1.39)	0.81 (1.00)	0.47 (.94)	0.81 (1.26)	0.85 (1.15)	1.09 (1.07)
*p*-value	0.05		0.052				0.593		
Crowns									
Mean (SD)	2.46 (2.48)	3.57 (2.50)	4.17 (2.13)	1.94 (2.52)	2.48 (2.59)	0.76 (1.85)	3.25 (2.60)	2.38 (2.59)	1.82 (1.91)
*p*-value	0.062		0.008 *				0.035 *		
			a vs. d: 0.008 *				a vs. c: 0.04 *		
Other									
Mean (SD)	0.88 (0.45)	0.79 (0.43)	0.79 (0.43)	0.94 (0.24)	0.81 (0.39)	0.41 (0.61)	1.03 (0.16)	0.81 (0.40)	0.71 (0.60)
*p*-value	0.177		<0.0001 *				<0.0001 *		
			a vs. d: 0.02 * b vs. d: <0.001 * c vs. d: 0.011 *				a vs. c: <0.0001 * b vs. c: 0.007 *		

* Statistically significant, † Mann–Whitney U test, ‡ Kruskal–Wallis test, ASD: Autism spectrum disorder. Different letters indicate statistically significant pairwise comparison (Dunn’s test).

**Table 7:** Association of ASD children’s demographic profile and medical status with the number of dental general anesthesia sessions delivered (*n* = 82).

DGA Sessions	Dentition Stage *n*% Mixed/Permanent	Gender *n*% Boys	ASD+ Comorbidities *n*%	Economic Level *n*%			ASD Severity *n*%		
				Low	Middle	High	Mild	Moderate	Severe
Nil	14(24.6)	11(19.3)	5(14.7)	8(23.5)	7(20)	3(23.1)	13(40.6)	3(9.1)	2(11.8)
One	5(8.8)	7(12.3)	4(11.8)	2(5.9)	6(17.1)	2(15.4)	5(15.6)	4(12.1)	1(5.9)
Two	36(63.2)	38(66.7)	24(70.6)	24(70.6)	20(57.1)	8(61.5)	14(43.8)	25(75.8)	13(76.5)
Three	2(3.5)	1(1.8)	1(2.9)	0(0)	2(5.7)	0(0)	0(0)	1(3)	1(5.9)
*p*-value	0.354	0.736	0.587		0.517			0.029 *	

* Statistically significant, chi-squared test, ASD: Autism spectrum disorder, DGA: dental general anesthesia.

## Data Availability

The datasets used and/or analyzed during the current study are available from the corresponding author upon reasonable request.
